# From pandemic to progress: maternal health resilience in the post COVID-19 era in Tamil Nadu, India

**DOI:** 10.1186/s12884-026-08704-2

**Published:** 2026-01-30

**Authors:** Kandaswamy Paramasivan, Ashwin Prakash

**Affiliations:** 1 Department of Management Studies, Indian Institute of Technology, Chennai, Madras India; 2 Department of Data Science and Artificial Intelligence, Indian Institute of Technology, Chennai, India; 3 Department of Computational Intelligence, SRM Institute of Science and Technology, Kattankulathur, Chengalpattu, India; 4 Moody’s Analytics, Bengaluru, Karnataka India

**Keywords:** Maternal mortality rate, Infant mortality rate, Home deliveries, Pandemic, C- section, Emergency medical services

## Abstract

**Background and Objectives:**

In India, like most countries, the COVID-19 pandemic in successive waves severely hampered the emergency medical services (EMS) and the government made prompt interventions, including substantial investments in both EMS and maternal health care immediately after the first wave. The study assessed variations in EMS efficiency and critical perinatal outcomes between the pre-pandemic era and the post pandemic-resilient phase in 2023 and 2024.

**Data and Methods:**

The study analysed the key EMS metrics based on the calls related to pregnancy, including call volume, response and transfer time, hospital handoff time and ambulance travel distance, and important maternal-newborn health outcomes such as maternal and neonatal mortalities, home deliveries, institutional childbirths, C-section deliveries, miscarriages and complicated vaginal births. The data relied upon encompasses the period from January 2017 to December 2024, including eight pandemic phases in 2020-22 and the resilient period of 2023 and 2024, obtained from the Tamil Nadu 108 Ambulance Control Room. A time series analysis method evaluated the EMS metrics in various pandemic phases; a statistical comparison was made with the pre-pandemic period for maternal-newborn outcomes. The appropriate effect size metric quantified the change in both analyses.

**Results:**

In the pandemic phases, despite an increase in pregnancy related call volume, the EMS metrics such as response times, transfer times and hospital handoff times witnessed notable improvement. The maternal and childbirth outcomes, especially in the post-pandemic and resilient phases during 2023-24, were markedly superior when compared with the corresponding period in the pre-pandemic era. In particular, the maternal mortality rate reduced by 19%, with 37 deaths per 100,000 live births. This is far lower than the national average of 97 deaths per 100,000 live births. Also, the rates of infant death, neonatal death, miscarriage, difficult vaginal births, and home births went down by 19.35%, 17.03%, 28.02%, 19.23%, and 36.05%, respectively.

**Conclusions:**

Government investments during the pandemic, along with the sustained focus on maternal health programmes, appear to have provided substantial support to pregnant women and newborns. The reproductive health of women in Tamil Nadu does not seem to have been undermined by the pandemic.

## Introduction

Globally, the COVID-19 pandemic, in its various waves, has severely disrupted healthcare services across different countries. Notably, the emergency medical services were overwhelmed with patients complaining of COVID-19 infection. It was very common to hear how important medical interventions were postponed or abandoned in view of the overcrowding of the hospitals by virus-infected patients fighting for their lives. In this context of large-scale disruption of maternal and obstetric care at several places worldwide, Tamil Nadu, the southernmost state in India, witnessed the COVID-19 pandemic in three distinct waves from March 2020 to March 2022, followed by the post-pandemic phase from April 2022 to December 2022 and finally the resilient recovery period from January to November 2023. The impact of the pandemic was more pronounced in maternal and child healthcare, as both pregnant women and newborn babies could not access the healthcare facilities, even for important diagnostic tests. The important emergency medical services (EMS) metrics the study investigates are response time, travel time, hospital handoff time, and the total distance travelled by ambulance.

At present, in Tamil Nadu, there are four tiers in the hospital system: the primary health centre, the community health centre, the district hospital, and the medical college hospital. The study included those pregnant women who had availed the 108 Ambulance of EMS. These women were provided with antenatal care that included multiple visits to designated medical facilities during their time of pregnancy under schemes like Pradhan Mantri Surakshit Matritva Abhiyan (PMSMA) and Tamil Nadu’s Dr Muthulakshmi Reddy Maternity Benefit Scheme and have been assessed with proper risk stratification. The present system that involves proactive planning assigns these women to appropriate delivery locations (PHC for normal cases and CEmONC – Comprehensive Emergency Obstetrics Neonatal Care – cases for Medical College Hospital). However, unanticipated complications arise, and emergency referrals during childbirth are critical. The referral system activates when specialist availability (e.g., obstetricians, anaesthetists, neonatologists) or diagnostic equipment and therapeutic infrastructure (e.g., ultrasound, blood bank, operative theatres, imaging facilities or advanced neonatal care equipment) is absent at the current facility, ensuring timely upward transport via 108 ambulances to higher-tier hospitals like medical colleges. Thus, the present referral system is designed to ensure rapid and appropriate escalation of care to higher-level institutions when such constraints arise, thereby preventing delays in emergency obstetric and neonatal interventions.

It was rather expected that there would be serious adverse consequences for outcomes relating to pregnant women and newborn babies, especially in the resilient phases that followed after the pandemic infection completely disappeared. This research seeks to assess the important outcomes measured through indices such as maternal mortality rate, neonatal mortality rate, infant mortality rate, institutional deliveries of babies, home deliveries, complicated vaginal child births, and C-section deliveries. The focus of the study was shifted from the pandemic years of 2020–2022 to the resilient span of 2023 and 2024. The overall aim of the research was to find whether the pandemic has had a lasting effect on the reproductive health outcomes, especially of mothers and infants. The entire health apparatus was crippled, causing serious disruption to routine health services that has prompted the reassessment of how EMS adjusted their operation and functioning to meet the needs of pregnant women and newborn babies. This research attempts to explore the link between the EMS efficacy and perinatal outcomes in the context of the disruption caused by the pandemic and contribute to policy directives and frontline practices for public health emergencies.

### Literature review

Globally, the COVID-19 pandemic, while it was a catastrophic event in human history, served as a test bed to critically evaluate the shortcomings in the healthcare system, including those concerning maternal and childcare, especially in the context of public emergency or calamity. It also paved the way for innovation, creativity and ingenuity to handle such crises. The present literature review not only highlights the adverse impact of the pandemic on Emergency Obstetric and Newborn Care (EmONC) in contextual places similar to India but also brings forth the scholarship concerning the novel ways in which governments, non-profits and other stakeholders adopted strategies to combat the pandemic and demonstrated resilience.

#### Adverse pandemic impact on maternal care and childbirth

During the pandemic crisis, pregnant women and their families had faced unmet needs, and inconsistent care revealed structural faultlines and institutional breakdowns [1]. Furthermore, there were huge disparities in access to and quality of health care worldwide, exacerbating equity concerns for marginalised social and economic communities [2]. Serious apprehensions and alarming news of the contagion forced people not to seek medical help, and overcrowding of hospitals induced hospital-avoiding behaviour [3–5]. Stay-at-home orders issued also severely restricted people’s mobility, including for reaching out to the hospitals, but many people could not afford to lose jobs, - a situation that contributed to depression [6, 7]. The scholarship from many regions consistently reports significant reductions in the consumption of vital services for pregnant women and reproductive health services during the pandemic. The research conducted in the Southeast region showed just how severe the disruption was: facility-based antenatal care and deliveries plummeted by 69.6% and 52.4%, respectively [8]. A systematic review and meta-analysis study from Ethiopia revealed that services related to family planning, prenatal care visitations and institutional baby deliveries significantly dropped by 26.2%, 19.30% and 12.82%, respectively [9]. Only 36% of the pregnant women in the Democratic Republic of Congo could complete the mandatory diagnostic and other procedures prior to delivery in view of financial hardship and vaccine hesitancy [10]. In Egypt, the severe disruptions in the healthcare system contributed directly to unintended pregnancies, associated health issues, and heightened overall risks [11]. 

In several settings, the pandemic-induced access barriers translated directly to concerning clinical outcomes. Due to COVID-19 infection among health care professionals led to closure of medical facilities negatively impacting maternal outcomes [12]. Brazil saw a big surge in maternal deaths and a 4.8% rise in stillbirths [13, 14]. The PregCovid registry in Maharashtra, India, reported additional challenges for mothers during the Delta wave, such as more preterm births and low birth weight [15]. Similarly, the severity of COVID-19 affected pregnant women in various ways. For instance, in Rio de Janeiro, maternal outcomes, such as ICU hospitalisations for critical concerns and mortality, were far more adverse during the Gamma Wave compared to the Delta Wave [16].

#### Innovative strategies – Better emergency care and maternal and childbirth outcomes

Despite these gloomy scenarios that were prevalent in most parts of the world, creative and committed people made a significant difference as they contributed to the strength in resilience in tackling the pandemic. For instance, in Malaysia, despite inordinate delays in maternal and child health services, immunisations for babies remained consistent [17]. A comprehensive analysis that involved a systematic review of literature databases showed that, besides increased support from various healthcare apparatuses and counselling on coping mechanisms, techniques to relieve stress should be part of prenatal care for better maternal and childbirth outcomes [18]. Further, another study found that maternal COVID-19 infection was associated with a heightened risk of obstetric complications, higher rates of C-section deliveries, and higher neonatal intensive care unit admissions; however, the neonatal outcomes, such as birthweight, did not significantly differ from those of babies born to mothers without COVID-19. This study indicates that systemic modifications in certain domains were advantageous [19].

When the situation is extraordinarily difficult and challenging, we need to think of extraordinary intervention. One of the innovative strategies was digital transformation in health care, besides using other simple, street-smart ideas to sustain care and treatment. The MomCare digital platform in Kenya helped people find care during lockdowns [20]. People would rather not move out in view of apprehension of infection, and similarly, the government wanted people to stay at home. Diligently in Indonesia, telehealth for consultations became popular [21], and smartphone apps helped people to monitor their health [22]. Iran set up a nationwide maternal health network to make sure that suggestions were followed [23]. Studies conducted in India revealed a 30% decline in institutional births in the northern region [24]. But government programmes like Janani Suraksha Yojana (JSY) acted as a safety net, and pregnant women utilised alternative ways to deal with stress [25]. The healthcare system in Tamil Nadu worked effectively, with a 62% rise in calls for ambulances for pregnant women [26] and 98% of women reporting they were delighted with the services [27]. In conclusion, the pandemic had a very varied effect on maternal health. It made inequalities worse, but it also led to quick new ideas. The literature emphasises that recovery and future readiness rely on the establishment of resilient systems that institutionalise effective adaptations—such as telehealth, community-based support [28], and enhanced emergency networks—while directly confronting the socioeconomic and geographic disparities that exacerbated outcomes for the most vulnerable [6, 12, 29].

## Data and method

### Data

In Tamil Nadu, one of the most populous states with an estimated population of 84 million people, residents can call 108 to get emergency medical services (EMS). There is a state control room for “108 Ambulance” EMS. The state is divided into 42 districts, each of which has its own public health administration that is overseen by the state headquarters. The present research utilises data from the State 108 EMS Registry, which caters to different types of callers from Tamil Nadu at no cost, including those related to pregnant women, nursing mothers, and newborn babies. When the calls are received at the control room, the dispatchers direct the right ambulance to the scene of the medical emergency, making sure that the ambulance is of the correct type and is properly equipped with medical supplies and staffed by para-medically trained personnel. The EMS team have positioned strategically, based on the historical data, the ambulances in key base areas so that people may quickly obtain emergency assistance. The study identifies many essential variables relevant to ambulance services: We call the time it takes for the ambulance to get to the emergency after the call was made as “response time”. The time it takes for the ambulance to take the patient from the scene of the emergency to the hospital is called transfer time. Lastly, the hospital handoff time is the time it takes for the ambulance to arrive at the hospital and for the paramedics in the ambulance to fully hand over the patient to the hospital’s emergency department. The ambulance travel distances are categorised into three segments: from the base to the emergency site, from the emergency site to the hospital, and from the hospital back to the base.

Emergency calls are categorized by their origin, with “Inter-Facility Transfer” calls used for transferring patients between medical facilities, and non-Inter-Facility Transfer calls typically originating from locations such as a pregnant woman’s home. Typically, ambulance efficiency can be gauged by response and transfer time; ambulance operational burden can be benchmarked by call volume and distance travelled ; and hospital preparedness for quick admission can be learned from hospital handoff time that excludes response and travel time.

The second set of statistics, which is the more essential one, has to do with perinatal outcomes. These statistics, which cover institutional and home births, complicated vaginal births, C-section deliveries, maternal mortality rates (MMR), neonatal and infant mortality rates for Tamil Nadu from 2013 to 2014 to 2023–2024, are particularly useful. The National Health Mission, the Department of Health and Family Welfare, and the Government of India provided the aforementioned details. This data includes all of Tamil Nadu’s citizens, including pregnant women and newborn babies who may have used either government or private hospital services or received care at home.

### Method

When looking at the effects of an intervention, randomised controlled trials are usually the most reliable way to do causal analysis. But for public health studies like the present one, it is impractical to randomly assign people to treatment or control groups. Counterfactual analysis may be a feasible approach; nevertheless, it is less relevant in this context due to the research’s emphasis on the pandemic’s effects, which persisted through post-pandemic and resilient phases over a duration exceeding two years. To solve this problem, the researchers used a variety of methodologies, including statistical methods like ARIMA, machine learning methods like generalised additive models, and advanced deep learning models like transformers. Despite experimenting with these techniques, errors encountered were beyond the acceptable limits in view of high volatility in the data, understandably due to large-scale disruptions caused by pandemic in a highly unpredictable manner.

The data for this study covers multiple time periods for Emergency Medical Services (EMS) and maternal outcomes. The EMS data starts in 2016, and the maternal data starts in 2013. The focus of the analysis is on the many phases of the pandemic, which are Wave-1, post-Wave-1, Wave-2, post-Wave-2, Wave-3, post-Wave-3, and the resilient period. Call volume, response time, transport time, hospital handover time, and total distance travelled by the ambulance for one call (TDT) are all EMS parameters. The study examines maternal outcomes, including institutional deliveries in government and private hospitals, home deliveries, miscarriages, caesarean sections, complicated vaginal births, maternal mortality, neonatal mortality, neonatal mortality within 7 days, and infant mortality. The study also figures out the rates of death for mothers and babies: the Maternal Mortality Rate, the Neonatal Mortality Rate, the Neonatal Mortality Rate-7 days, and the Infant Mortality Rate.

EMS data is analyzed as daily mean values, while maternal outcomes are considered as monthly averages. To compare pandemic-phase data with the pre-pandemic period, for instance, the response time during Wave-1 (March 23, 2020, to September 30, 2020) is compared with the pre-pandemic data from March 23 to September 30 in 2016, 2017, 2018, and 2019. This results in two distributions with 191 data points each: one for the pandemic period and the other for the corresponding pre-pandemic days. A basic comparison of the means of different distributions might miss the differences in the data, which could lead to wrong conclusions. The study compares the complete distribution of each statistic, whether time metric or distance variable, over various phases of the pandemic with their corresponding pre-pandemic distributions.

First, the data’s normality is checked. If the distribution is normal, a t-test is administered to see if there are significant variations between the two periods. The Wilcoxon test is employed to find statistical differences for non-Gaussian distributions. In our study, most distributions violate the normality assumption. Cliff’s Delta is used to find the effect size. The Cliff’s Delta number shows how much things have changed, whether it’s a small, medium, or large or very large. It can be anywhere from − 1 to + 1. For normal distributions, Cohen’s D is the metric for effect size.

## Results

The first section presents the results of EMS response, evaluated using five metrics: Call Volume, Response Time, Transfer Time, Hospital Handover Time, and Total Distance Travelled. This is followed by a discussion of maternal outcomes, which are assessed using twelve different metrics. Inter-facility calls involve transfer from one medical facility to another, whereas non-interfacility calls are from the home of a pregnant woman or the scene of an emergency to the appropriate medical facility.

### EMS-Metrics

There were four main trends in the number of non-inter-facility transfer calls. First, there was a moderate drop in non-inter-facility transfer calls during Wave-1 and post-Wave-1 (with a medium effect size). The second trend was a meagre escalation during Wave 2, which was the most disastrous phase of the pandemic and had the highest number of COVID-19 cases and fatalities. Third, after Wave-2, Wave-3, and post-Wave-3, there was a steady rise in call volume, with a substantial impact size observed. Finally, the fourth trend was a sharp decline in call volume during the resilient period, marked by a significant effect size reduction. As for inter-facility transfer call volume, there was a continuous upward surge across most pandemic phases, with effect sizes ranging from large to very large, except during Wave-2, where the increase was minimally significant. The distinct Wave-2 phase only showed an insignificant rise in Inter-Facility Transfer calls, standing out from the other periods. (See Table-1)

**Table 1:** Percentage difference between actual and Reference (Pre-Pandemic) total calls, Inter-Facility transfer calls & Non- Inter-Facility transfer calls related to pregnancy along with effect size (Cliffs Delta; cohen’s D) and respective confidence intervals during various phases of lockdowns in all three waves in 2020–2022 and resilience period in 2023 and 2024 in Tamil Nadu

Period	Actual	Reference	% Change	Avg. Ref. Daily Call	Avg Actual Daily Call	*N*- test Actual	*N*- test- Pre-Pandemic	Wilcoxon Test	Effect Size	Confidence Interval - Effect Size
Inter-Facility Transfer										
Wave 1	96,256	88,397	8.89	460	501	< 0.001	< 0.001	< 0.001	0.63 Medium*	[0.43, 0.83]
Post Wave 1	99,149	78,012	27.09	429	545	< 0.001	< 0.001	< 0.001	0.49 large	[0.38, 0.59]
Wave 2	92,299	85,709	7.69	468	504	< 0.001	< 0.001	< 0.001	0.21 small	[0.08, 0.32]
Post Wave 2	49,946	44,023	13.45	543	617	0.446	< 0.001		0.97 Large*	[0.63, 1.29]
Wave 3	39,622	34,184	15.91	422	489	0.893	0.879		1.05 Large*	[0.74, 1.36]
Post Wave 3	165,250	137,063	20.57	465	560	< 0.001	< 0.001	< 0.001	0.57 large	[0.49, 0.63]
Resilience Period	107,256	153,684	-30.21	460	321	< 0.001	< 0.001	< 0.001	-0.73 large	[-0.78, -0.68]
Non- Inter-Facility Transfer										
Wave 1	41,588	48,606	-14.44	253	217	< 0.001	< 0.001	< 0.001	-0.45 medium	[-0.55, -0.32]
Post Wave 1	29,310	38,835	-24.53	213	189	< 0.001	< 0.001	< 0.001	-0.36 medium	[-0.45, -0.21]
Wave 2	46,161	38,602	19.58	211	252	< 0.001	< 0.001	< 0.001	0.08 negligible	[-0.06, 0.2]
Post Wave 2	26,781	18,588	44.08	229	331	< 0.001	< 0.001	< 0.001	0.61 large	[0.44, 0.74]
Wave 3	21,288	14,941	42.48	184	263	< 0.001	< 0.001	< 0.001	0.66 large	[0.51, 0.78]
Post Wave 3	89,938	70,420	27.72	239	305	< 0.001	< 0.001	< 0.001	0.36 medium	[0.27, 0.44]
Resilience Period	51,832	77,476	-33.10	232	155	< 0.001	< 0.001	< 0.001	-0.64 large	[-0.7, -0.57]

Nearly all time-based metrics, including Response Time, Transfer Time and Hospital Handover Time, saw a significant reduction during the pandemic period, from post-Wave-1 to Resilience Period for both Inter-Facility Transfer and non-inter-facility transfer calls. However, the decrease was more pronounced during the phases of Wave-2, post-Wave-2, Wave-3, and post-Wave-3, while the declines in Wave-1, post-Wave-1, and RP were less substantial. These reductions were compared to corresponding pre-pandemic periods. Overall, the declines in time metrics were largely consistent across all phases, with only slight differences emerging during the Wave-3, post-Wave-3, and Resilience Period phases. There was significant reduction in response time, transfer time and hospital handoff time in the former category compared with latter. On the other hand, distance metric, for the entire period from Wave-1 to Wave-3, in the former category there was an increase, while for the latter there was decrease. As for Inter-Facility Transfer calls, there was a continuous upward surge across most pandemic phases, with effect sizes ranging from large to very large, except during Wave-2, where the increase was minimally significant. The distinct Wave-2 phase only showed an insignificant rise in Inter-Facility Transfer calls, standing out from the other periods (See Tables 2, 3, 4 and 5).

**Table 2:** Percentage difference between Actual and Reference (Pre-Pandemic)Inter-Facility transfer and Non-Inter-Facility transfer response time (Mins) for pregnancy related calls with effect size (Cliffs Delta; cohen’s D) and respective confidence intervals during various phases of lockdowns in all three waves in 2020–2022 and resilience period in 2023 and 2024 in Tamil Nadu

Period	Actual	Reference	% Change	*N*- test Actual	*N*- test- Predicted	Wilcoxon Test	Effect Size	Confidence Interval - Effect Size
Inter-Facility Transfer								
Wave 1	22.03	21.91	0.56	< 0.001	< 0.001		0.08 Very small*	[-0.12, 0.28]
Post Wave 1	15.98	20.74	-22.95	< 0.001	< 0.001	< 0.001	-0.71 large	[-0.78, -0.62]
Wave 2	14.68	20.58	-28.69	< 0.001	< 0.001	< 0.001	-0.82 large	[-0.88, -0.75]
Post Wave 2	14.66	22.58	-35.08	0.767	0.5		-4.14 Very Large*	[-4.66, -3.6]
Wave 3	14.93	19.98	-25.28	0.44	0.739		-3.0 Very Large*	[-3.4, -2.6]
Post Wave 3	15.36	20.96	-26.70	< 0.001	< 0.001	< 0.001	-0.97 large	[-0.98, -0.95]
Resilience Period	15.81	20.85	-24.21	< 0.001	< 0.001	< 0.001	-0.94 large	[-0.96, -0.92]
Non- Inter-Facility Transfer								
Wave 1	23.98	24.26	-1.18	< 0.001	< 0.001	< 0.001	-0.06 negligible	[-0.17, 0.06]
Post Wave 1	18.09	23.27	-22.27	< 0.001	< 0.001		-0.5 large	[-0.64, -0.42]
Wave 2	12.44	22.27	-44.14	< 0.001	< 0.001	< 0.001	-0.72 large	[-0.79, -0.64]
Post Wave 2	11.28	23.85	-52.70	< 0.001	< 0.001	< 0.001	-0.85 large	[-0.92, -0.75]
Wave 3	12.28	22.84	-46.24	< 0.001	< 0.001	< 0.001	-0.84 large	[-0.92, -0.72]
Post Wave 3	12.30	21.87	-43.76	< 0.001	< 0.001	< 0.001	-0.8 large	[-0.85, -0.74]
Resilience Period	11.86	22.04	-46.19	< 0.001	< 0.001	< 0.001	-0.81 large	[-0.85, -0.75]

**Table 3:** Percentage difference between Actual and Reference ( Pre-Pandemic) Non-Inter-Facility transfer and Inter-Facility transfer travel time (Mins) with effect size (Cliffs Delta; cohen’s D) and respective confidence intervals during various phases of lockdowns in all three waves in 2020–2022 and resilience period in 2023 and 2024 in Tamil Nadu

Period	Actual	Reference	% Change	*N*- test Actual	*N*- test- Predicted	Wilcoxon Test	Effect Size	Confidence Interval - Effect Size
Inter-Facility Transfer								
Wave 1	55	59.54	-7.63	< 0.001	< 0.001	< 0.001	-0.84 large	[-0.89, -0.78]
Post Wave 1	53	57.00	-7.82	< 0.001	< 0.001	< 0.001	-0.84 large	[-0.88, -0.77]
Wave 2	53	56.02	-5.43	< 0.001	< 0.001	< 0.001	-0.79 large	[-0.85, -0.72]
Post Wave 2	53	56.02	-5.39	< 0.001	< 0.001	< 0.001	-0.86 large	[-0.92, -0.75]
Wave 3	55	56.03	-1.83	0.003	< 0.001	< 0.001	-0.63 large	[-0.75, -0.48]
Post Wave 3	55	56.50	-2.65	< 0.001	< 0.001	< 0.001	-0.46 medium	[-0.54, -0.37]
Resilience Period	55	56.50	-2.65	< 0.001	< 0.001	< 0.001	-0.35 medium	[-0.43, -0.26]
Non-Inter-Facility Transfer								
Wave 1	35	33.03	5.96	< 0.001	< 0.001	< 0.001	0.26 small	[0.14, 0.37]
Post Wave 1	29	30.24	-4.08	< 0.001	< 0.001		-0.24 small	[-0.37, -0.12]
Wave 2	25	30.02	-16.72	< 0.001	< 0.001	< 0.001	-0.45 medium	[-0.55, -0.34]
Post Wave 2	24	32.00	-25.00	< 0.001	< 0.001	< 0.001	-0.68 large	[-0.79, -0.54]
Wave 3	26	31.02	-16.18	< 0.001	< 0.001	< 0.001	-0.48 large	[-0.62, -0.3]
Post Wave 3	26	32.00	-18.75	< 0.001	< 0.001	< 0.001	-0.49 large	[-0.56, -0.4]
Resilience Period	27	31.99	-16.27	< 0.001	< 0.001	< 0.001	-0.44 medium	[-0.52, -0.36]

**Table 4:** Percentage difference between Actual and Reference (Pre-Pandemic) Non-Inter-Facility transfer and Inter-Facility transfer hospital handoff time (Mins) with effect size (Cliffs Delta; cohen’s D) and respective confidence intervals during various phases of lockdowns in all three waves in 2020–2022 and resilience period in 2023 and 2024 in Tamil Nadu

Period	Actual	Reference	% Change	*N*- test Actual	*N*- test- Predicted	Wilcoxon Test	Effect Size	Confidence Interval - Effect Size
Inter-Facility Transfer								
Wave 1	15.00	15.03	-0.19	< 0.001	< 0.001	< 0.001	-0.69 large	[-0.76, -0.6]
Post Wave 1	15.03	16.02	-6.14	< 0.001	< 0.001	< 0.001	-0.32 small	[-0.43, -0.2]
Wave 2	15.00	17.02	-11.85	< 0.001	< 0.001	< 0.001	-0.97 large	[-0.99, -0.95]
Post Wave 2	15.00	18.02	-16.74	< 0.001	< 0.001	< 0.001	-0.96 large	[-0.98, -0.9]
Wave 3	15.58	17.02	-8.42	< 0.001	< 0.001	< 0.001	-0.63 large	[-0.75, -0.48]
Post Wave 3	17.01	18.00	-5.51	< 0.001	< 0.001	< 0.001	-0.33 small	[-0.41, -0.23]
Resilience Period	19.00	18.00	5.56	< 0.001	< 0.001	< 0.001	0.3 small	[0.21, 0.38]
Non-Inter-Facility Transfer								
Wave 1	14.03	14.00	0.18	< 0.001	< 0.001	< 0.001	0.01 negligible	[-0.1, 0.13]
Post Wave 1	13.02	14.99	-13.15	< 0.001	< 0.001		-0.31 small	[-0.45, -0.21]
Wave 2	11.00	14.58	-24.53	< 0.001	< 0.001	< 0.001	-0.64 large	[-0.71, -0.54]
Post Wave 2	11.71	15.00	-21.94	< 0.001	< 0.001	< 0.001	-0.73 large	[-0.83, -0.61]
Wave 3	12.23	15.00	-18.44	< 0.001	< 0.001	< 0.001	-0.55 large	[-0.68, -0.39]
Post Wave 3	15.00	15.00	0.00	< 0.001	< 0.001	< 0.001	-0.19 small	[-0.28, -0.1]
Resilience Period	15.00	15.00	0.00	< 0.001	< 0.001	< 0.001	-0.07 negligible	[-0.15, 0.02]

**Table 5:** Percentage difference between Actual and Reference (Pre-Pandemic) Non-Inter-Facility transfer and Inter-Facility transfer total distance travelled (in Kms) with effect size (Cliffs Delta; cohen’s D) and respective confidence intervals during various phases of lockdowns in all three waves in 2020–2022 and resilience period in 2023 and 2024 in Tamil Nadu

Period	Actual	Reference	% Change	*N*- test Actual	*N*- test- Pre-Pandemic	Wilcoxon Test	Effect Size	Confidence Interval - Effect Size
Inter-Facility Transfer								
Wave 1	60	57	5.26	< 0.001	< 0.001		1.15 Large*	[0.94. 1.37]
Post Wave 1	59	56	5.36	< 0.001	< 0.001	< 0.001	0.35 medium	[0.23, 0.46]
Wave 2	59	56	5.36	< 0.001	< 0.001	< 0.001	0.56 large	[0.46, 0.65]
Post Wave 2	57	55	3.64	0.266	< 0.001		0.54 Medium*	[0.21, 0.85]
Wave 3	57	56	1.79	0.23	< 0.001	< 0.001	0.16 small	[0.01, 0.33]
Post Wave 3	56	56	0	< 0.001	< 0.001	< 0.001	-0.16 small	[-0.25, -0.07]
Resilience Period	55.5	56	-0.89	< 0.001	< 0.001	< 0.001	`-0.01 negligible	[-0.08, 0.09]
Non-Inter-Facility Transfer								
Wave 1	38	31	22.58	< 0.001	< 0.001	< 0.001	0.74 large	[0.65, 0.81]
Post Wave 1	31	31	0	< 0.001	< 0.001		-0.07 negligible	[-0.06, 0.2]
Wave 2	25	30	-16.67	< 0.001	< 0.001	< 0.001	0.2 small	[-0.32, 0.08]
Post Wave 2	23	32	-28.13	< 0.001	< 0.001	< 0.001	-0.52 large	[-0.66, -0.34]
Wave 3	25	32	-21.88	< 0.001	< 0.001	< 0.001	-0.44 medium	[-0.58, − 0.26]
Post Wave 3	23	33	-30.3	< 0.001	< 0.001	< 0.001	-0.58 large	[-0.66, -0.5]
Resilience Period	23	33	-30.3	< 0.001	< 0.001	< 0.001	-0.51 large	[-0.58, -0.44]

### Maternal and childbirth outcomes

Among various maternal and childbirth outcome metrics, home deliveries, miscarriages, complicated vaginal births, neonatal mortality, maternal mortality rate and neo-natal morality showed more significant changes than others. The most notable impact of the pandemic was the surge in home deliveries during critical phases like post-Wave-1, Wave-2, and post-Wave-2, with increases of 66.3%, 15.9%, and 30.7% respectively, compared to pre-pandemic periods. Maternal morality and maternal mortality rate saw a dramatic rise of 98.5% and 109.2% during Wave-2, and Wave-3 experienced significant increases of 53.7% and 60.4% respectively, compared to pre-pandemic times. Neo-natal mortality and Neo-natal mortality within 7 days, consistently rose during the pandemic, though only moderately, hovering around a 10% increase. The infant mortality rate followed a similar trend, with slightly lower increases.

The other metrics, such as institutional births in both private and government sectors, as well as C-section deliveries, exhibited only marginal fluctuations in percentage across the different pandemic and post-pandemic phases. In contrast, both Infant Mortality and Infant Mortality Rate showed a moderate increase throughout the pandemic phases when compared to the corresponding pre-pandemic periods, except during the resilient period, where there was a reduction of 14% and 9.7%, respectively.

**Fig. 1:**
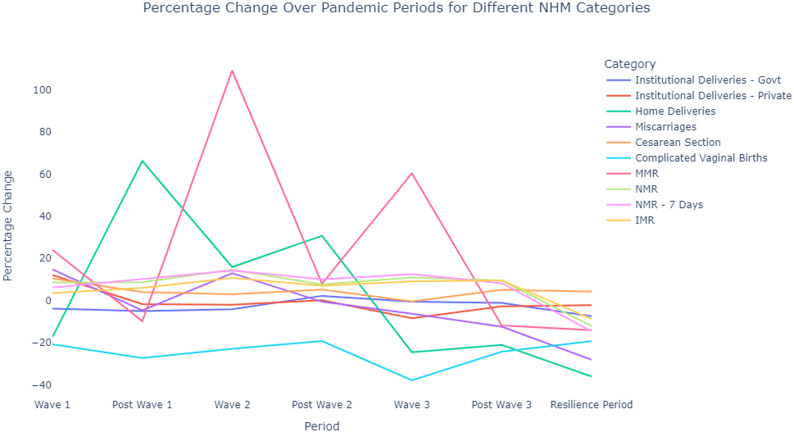
Shows the percentage change in various maternal and childbirth outcomes in Tamil Nadu, India during the seven pandemic phases compared with the corresponding pre-pandemic phase as provided by National Health Mission (NHM)

**Fig. 2:**
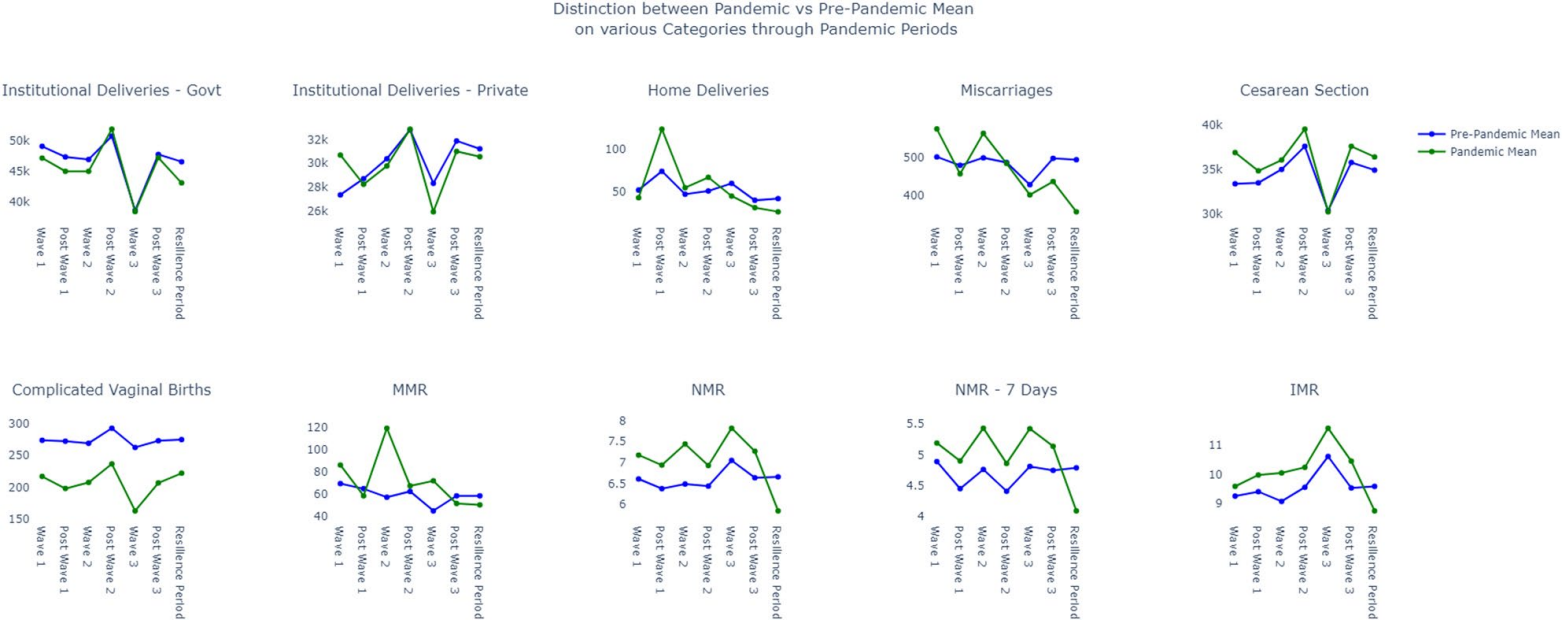
Illustrates the variation in maternal and childbirth outcomes across seven distinct pandemic phases, compared to pre-pandemic periods. The X-axis denotes the time windows during the pandemic phases, while the Y-axis represents the mean number of maternal and childbirth cases for specific categories

In contrast, complicated vaginal births sharply declined across all pandemic phases, with percentage drops ranging from 19.2% to 37.8%. During the various phases of the pandemic, most metrics, except for complicated vaginal births, saw moderate to significant increases. However, in the resilient period, all metrics experienced a downward trend compared to pre-pandemic levels, with home deliveries decreasing by 36.1%, maternal complications by 28.1%, complicated vaginal births by 19.2%, maternal mortality (MM) by 19%, and neonatal mortality (NM) by 17%. (See Table 6). Figure 1 illustrates that most of the lines representing most metrics converge well below the zero mark, emphasizing the significant improvements achieved. Figure 2 illustrates, across ten distinct panels, the trends observed in maternal outcome metrics throughout seven different phases, covering both the pandemic and post-pandemic periods. Notably, during the resilient phase, indices such as C-Section rates, maternal mortality rates (MMR), infant mortality rates (IMR), neonatal mortality rates (NMR), and NMR-7 were significantly lower than the rates observed in the pre-pandemic period.

**Table 6:** Shows the percentage change in the different maternal and childbirth outcomes during the various phases of pandemic and post pandemic when compared with the corresponding pre-pandemic period

Category	Wave-1	Post-Wave-1	Wave-2	Post-Wave-2	Wave-3	Post-Wave-3	Resilient Period
% Change	% Change	% Change	% Change	% Change	% Change	% Change	% Change
Institutional Deliveries - Govt	-3.9	-4.9	-4.1	2.3	-0.5	-1.1	-7.4
Institutional Deliveries - Private	12.2	-1.7	-2.0	0.2	-8.4	-2.8	-2.1
Home Deliveries	-17.3	66.3	15.9	30.7	-24.5	-21.0	-36.1
Miscarriages	14.8	-4.7	13.0	-0.5	-6.2	-12.4	-28.0
Cesarean Section	10.5	4.0	3.0	5.2	-0.4	5.1	4.3
Complicated Vaginal Births	-20.7	-27.3	-22.9	-19.2	-37.8	-24.3	-19.2
Maternal Mortality	26.3	-12.9	98.5	8.6	53.7	-13.0	-19.0
Neonatal Mortality	10.6	3.8	11.4	9.3	6.9	7.3	-17.0
Neonatal Mortality − 7 Days	8.1	5.2	11.0	11.6	8.5	6.2	-19.3
Infant Mortality	5.6	0.6	7.3	8.6	5.1	7.3	-14.0
MMR	24.1	-9.8	109.2	8.0	60.4	-11.8	-14.0
NMR	8.6	8.7	14.7	7.6	11.0	9.5	-12.1
NMR − 7 Days	6.2	10.2	14.3	10.1	12.6	8.2	-14.5
IMR	3.6	6.0	10.7	7.1	9.1	9.6	-8.7

## Discussion

This study investigates the influence of the COVID-19 pandemic on maternal and child health outcomes, specifically during the resilient phase of 2023–2024, while evaluating the significance of government measures in healthcare systems, notably concerning emergency medical services (EMS).The primary objective of this research is to determine whether the pandemic has had any medium-term impact on various EMS efficiency metrics—such as response time, travel time, hospital handoff time, and total distance travelled by ambulance—and consequently on maternal and childbirth outcomes, particularly regarding the reproductive health of women in Tamil Nadu. Therefore, our investigation focuses on the resilient period, which is defined as the time immediately following wave 3, the last wave of the COVID-19 pandemic. When wave-3 and the post-wave-3 period ended in the year 2022, the years 2023 and 2024 constituted the resilient phase. Literature supports that longer response time and travel time [30] result in less than desirable maternal and neonatal outcomes, and improper or poorer antenatal and perinatal care is likely to result in low birthweight children and a higher likelihood of mortality [31, 32].

Before getting into the results, it is pertinent to explain some aspects of the pandemic waves. The wave-2 was more harmful and disastrous, as it spread more rapidly both temporally and spatially than wave-1 and wave-3. Furthermore, the rate of the number of persons infected and who succumbed to COVID-19 was highest in this Wave-2 [33, 34]. The government, quickly learning from wave 1, had invested sufficiently in improving the health infrastructure, particularly keeping COVID-19 infection in mind. On the EMS front, the ambulance fleet was augmented [35], and a large number of doctors and paramedical staff were hired for EMS and regular hospital [36].

During the pandemic, call volumes for emergency services increased due to the emergence of new COVID-19 variants. There was an increase in inter-facility transfer calls connected to pregnancy. This boost was probably because some hospitals were designated only for COVID-19 patients and other facilities provided dedicated perinatal care to pregnant women in spaces separate from COVID-19 patients [37]. During the pandemic phases from Wave-1 to post-Wave-3, the entire healthcare industry whether private or public witnessed surge in call volume in view of diversion of all efforts to tackle pandemic, however in the resilient phase, there was return to normalcy as indicated by reduced call volume in comparison with the pre-pandemic levels. Hospital avoidance behavior noticed across all categories of callers including pregnant women during the more acute pandemic phases namely Wave-1, post-Wave-1 and Wave-2 and the stay-at-home orders contributed to reduced calls among pregnant women [38–40]. Immediately after Wave-1, the reduced EMS time metrics such as response time, travel time and hospital handoff time may be likely to have been the effect of improved hospital infrastructure, recruitment of different categories of health care professionals including doctors and augmented fleet strength of ambulance [41].

Home deliveries increased sharply during most pandemic phases, especially during the more severe waves, likely due to hospital avoidance. This trend was more pronounced early in the pandemic and during the more severe phases, when compliance with COVID-19 protocols was high. However, home delivery numbers continued to rise during later phases, possibly due to lingering pandemic-related concerns. Alongside the increase in home deliveries, institutional births in both public and private hospitals moderately declined. This decrease may have also contributed to the reduction in complicated vaginal births, as fewer institutional births occurred during the pandemic compared to pre-pandemic phases.

Even though the government worked to improve EMS and other maternal health care, MMR, the number of maternal deaths (death of a woman while pregnant or within 42 days of termination of pregnancy) per 100,000 live births did not come down. During Wave-2 and Wave-3, the MMR in fact, escalated. However, during the resilient period, there was a huge improvement in important maternal and neonatal health indicators. For example, home deliveries dropped by 36.1%, miscarriages dropped by 28.1%, complicated vaginal births dropped by 19.2%, maternal mortality dropped by 19%, and neonatal mortality dropped by 17%. The rise in maternal mortality during Wave-2 is linked to fewer institutional births and more home births. This is because home births are more likely to lead to maternal and newborn fatalities because of a lack of proper medical treatment during labour. Also, COVID-19 infections may have made problems with maternal health worse, which may have led to more deaths.

Although mortality-related indicators were elevated throughout the pandemic, they were not markedly worse in comparison to worldwide impacts. During the resilient era, all indicators, including maternal mortality, neonatal mortality, maternal mortality rate, newborn mortality rate, and neonatal mortality within 7 days, exhibited significant enhancements beyond pre-pandemic values. These improvements may be due to better healthcare infrastructure, better prenatal care, better emergency referrals, and other things that helped. The study acknowledges that other variables may have impacted these results and recommends further examination.

### Integrated analysis: operational efficiency and clinical rationale

This study integrates two parallel analyses: examination of time series data of daily average of key performance indicators of EMS and a descriptive statistical evaluation of reproductive, maternal, neonatal, and child health (RMNCH) outcomes. The aim is to analyse maternal care throughout the post-wave recovery and resilience phase following the third COVID-19 wave. The comparative statistical estimation of distributions reveal that, during this period, response time and travel time decreased significantly and moderately, respectively, compared to pre-pandemic trends, indicating enduring enhancements in emergency service delivery. These trends are likely a result of pandemic-era investments in emergency medical services infrastructure, such as adding more ambulances, hiring more paramedics, and making referral and dispatch systems stronger. All of these things are known to make it easier to receive timely emergency obstetric care ( [42–44]). Because pregnancy problems can happen at any time, emergency obstetric care is still the best way to lower the number of maternal fatalities around the world caused by these complications [45]. Simultaneously, a comparison of maternal and neonatal outcomes with the three-year pre-pandemic baseline indicated significant decreases in home deliveries, miscarriages, and complicated vaginal births, accompanied by enhancements in maternal mortality, maternal mortality ratio, and newborn mortality. While the temporal alignment of ameliorated EMS performance and favourable maternal–neonatal outcomes is evident, this study does not infer a causal relationship between reductions in time-based EMS metrics and outcome improvements. Nonetheless, present research indicates that prompt access to proficient obstetric and neonatal care diminishes the risk of intrapartum problems, including postpartum haemorrhage, sepsis, and delivery asphyxia [46]. These results show that the government interventions are keeping pace with the shifting health demands, higher public expectations, and new health goals [47]. In order to exactly pinpoint how the investments made in the health care by the government and other stakeholders and the newer policies implemented and perceived at both provider and community levels resulted in enhanced maternal and childbirth outcomes, it necessitates a deeper qualitative inquiry, including structured interviews, focus group discussions and adopting a mixed-method approach to complement the quantitative findings.

### Defining the scope of referrals in this study

This section continues the brief description of Tamil Nadu’s maternal and childbirth care referral system. It is clarified that the referral system adopted in 108 Ambulance EMS and the various hospitals under the Department of Health and Family Welfare is based on the system’s escalation protocol, which is primarily structural, logistical, and facility-readiness rather than strictly clinical. The government schemes ensure that all pregnant women are systematically screened, mandatory diagnostic procedures are done, and risk-stratified and pre-designated child delivery is done at an appropriate medical facility during the antenatal period; however, unanticipated complications may arise. In these cases, the decision to make an emergency referral is often not based on a sudden worsening of the patient’s condition but on the health facility’s pre-set limits, such as the availability of specialist healthcare professionals (anaesthetists, paediatricians, or neonatologists) or the lack of necessary diagnostic and therapeutic infrastructure and life-support equipment. This proactive logistical manoeuvre involves placing the patient in a facility that has comprehensive emergency obstetric and newborn care capabilities, thereby mitigating the risk of facility-level delays. This referral architecture addresses the resources gap and infrastructural constraints that are present in the existing government health care facilities. Consequently, our data captures the outcome of this logistical protocol. To properly assess the appropriateness of each referral—ascertaining whether the patient’s condition warranted the heightened degree of therapy administered—requires a specific methodological approach that encompasses clinical audit and case-note analysis. This offers a significant pathway for future study to enhance the operational findings presented above.

### Novelty and contribution of the analysis

This research is not conforming to widely available literature that speaks on the disruptions in medical service and disastrous effect on the health of the population. The comprehensive analysis of EMS Key Performance Indicators (KPIs) alongside the perinatal outcomes during the various pandemic phases, including the post-pandemic resilient period. Additionally, this research inquiry is thorough, examining the pandemic wave and its phase-wise disaggregation concerning both system performance and population health. These results showed an unanticipated improvement in operations, particularly regarding shorter response, travel, and hospital handoff times during periods of severe pandemic wave intensity, when COVID-related mortality was at its peak and healthcare facilities were overwhelmed. The key conclusion is the contrast between this logistical improvement and steady maternal health outcomes at the population level, rather than outcomes that have gotten much worse. This co-occurrence contradicts the traditional narrative of systemic breakdown, highlighting how targeted efforts inside the pre-hospital emergency care subsystem may have acted as a mitigating factor, offering a nuanced view of health system resilience.

### Limitations of the study

The authors recognise that the observed enhancements in maternity and delivery outcomes, particularly in the post-pandemic and resilient years, may be attributable to many exogenous factors beyond the scope of the government’s initiatives for improving medical facilities.

The other problem is that the study only uses quantitative data. While these methods are rigorous and reliable, they may not fully reflect the real situation, especially since medical disorders and maternal healthcare problems, including death, may not be reported or may be underreported. Furthermore, the study is solely focused on the state of Tamil Nadu, India, limiting the generalisability of the findings to other regions, as disparities in population health indicators, the balance between public and private healthcare services, and demographic-specific maternal concerns vary across different areas.

The study is limited to the immediate post-pandemic period (2023–2024), potentially failing to fully elucidate the long-term impacts of the pandemic on maternal health outcomes.

The study also lacked empirical evidence in establishing a causal relationship between data from EMS and NHM. This has beenfurther explained under Integrated Analysis: Operational Efficiency and Clinical Rationale section.

### Future research scope

Building on the present research, which is based on mapping the aggregative EMS performance metric to overall perinatal outcomes, future research should investigate patient-level and event-sequence dynamics that link response time, travel time, hospital handoff time, and distance travelled with specific maternal and neonatal outcomes. More granular study could be attempted to show how incremental improvements in the EMS process translate into sustained outcome gains during various stages of pregnancy and childbirth, including antenatal emergencies, intrapartum care and early neonatal periods. Furthermore, studies may be taken up to examine the facility-level preparedness indicators, referral decision thresholds, and spatial accessibility of resources to study system interactions within tiered hospital networks.

The differential findings between interfacility transfer and non-interfacility transfer motivate us to explore the long-term effectiveness of redesigning hospital work organisations to promote multi-professional teamwork and systemic accountability to reduce intra-hospital delays.

While this study did not embark on the digital innovations introduced during the pandemic elsewhere, such as the MomCare digital platform, telehealth, and smartphone applications to monitor health, further research requires longitudinal investigation to ensure healthcare system resilience.

One of the serious limitations of the study was not being able to find the exact causal link between the EMS efficiency and better perinatal outcomes. The future study can be on the qualitative study incorporating the perspectives of healthcare professionals in EMS and hospitals to unlock the contribution of any external parameter to the observed health comes from Tamil Nadu.

## Conclusions

The study highlights significant improvements in maternal health outcomes during various stages of the pandemic and post-COVID-19, particularly during the resilient period in 2023 and 2024, with a focus on government interventions. The study examined essential emergency medical service (EMS) indicators and maternal health outcomes across different stages of the pandemic and the subsequent resilience period.

The pandemic, especially in wave − 2, the most deleterious phase, had taken a serious toll on lives and raised serious health concerns for several people of different ages and genders. In most phases of the pandemic in three waves, EMS efficiency improved a lot, with shorter response times, transfer times, and hospital handoff times. Even though the number of calls increased, there was a substantial increase in the caseload for ambulance services, and EMS efficiency only improved. Even though the pandemic caused challenges, the years 2023 to 2024 saw a big improvement in maternal outcomes.. Marking a key phase of the resilient period, the Maternal Mortality Ratio (MMR) fell to 37 deaths per 100,000 live births—a substantial 19% decrease from the pre-pandemic baseline.

Notably, the maternal mortality rate in the state of Tamil Nadu fell to 24 in June 2024, considerably lower than the national average and that of other emerging countries, for which the rate is typically around 100. Furthermore, other maternal and childbirth outcomes, such as neonatal mortality, infant mortality, home deliveries, and complicated vaginal births, declined sharply in the resilient period when compared with the mean pre-pandemic levels of yesteryears.

While our investigation cannot be definitive that improved emergency medical service efficiency caused better maternal outcomes, their co-occurrence suggests that systematic adaptations would likely have played an important role in mitigating adverse pandemic effects on the reproductive health of women in Tamil Nadu. To confirm the causal link, future qualitative research employing structured interviews and focus group discussions with experts, including doctors, paramedics, and other stakeholders, such as EMS service personnel and families of the callers related to pregnancy and childbirth.

## Data Availability

The data that support the findings of this study are not openly available due to reasons of sensitivity and are available from the corresponding author upon reasonable request .

## Abbreviations

C: Sections–Caesarean Sections
EMS: Emergency Medical Services
IMR: Infant Mortality Rate
MM: Maternal Mortality
MMR: Maternal Mortality Rate
NM: Neonatal Mortality
NM: 7 Neonatal Mortality within 7 days
NMR: Neonatal Mortality Rate
NMR: 7–Neonatal Mortality Rate within 7 days
